# Venous thrombosis in neonates

**DOI:** 10.12703/r/10-20

**Published:** 2021-02-25

**Authors:** Mihir D Bhatt, Anthony KC Chan

**Affiliations:** 1 Division of Pediatric Hematology/Oncology, Department of Pediatrics, McMaster Children's Hospital, McMaster University, Hamilton, ON, Canada

**Keywords:** Neonate, thrombosis

## Abstract

The incidence of thrombotic disorders in neonates and children is increasing with advances in diagnostic modalities, supportive care, and management of many health conditions. The developing coagulation system, need for intensive care, including catheterization, and co-morbid conditions are responsible for the relatively high risk of thrombosis in neonates compared to older children. This review addresses the advances over the last 3 years in neonatal thrombosis, with a focus on epidemiology, cerebral sinovenous thrombosis (CSVT), renal vein thrombosis (RVT), and portal vein thrombosis (PVT). The incidence of neonatal thrombosis in the contemporary era is reported to be 6.9–15 per 1,000 neonatal intensive care unit (NICU) admissions, compared to 2.4 per 1,000 NICU admissions reported in older registry data. The majority of recently published studies are small, retrospective, and from single centers, albeit they emphasize the need for definitive data to support the efficacy and safety of anticoagulation therapy (ACT) in the management of CSVT, RVT, and PVT. We highlight two important international initiatives geared towards improving the evidence base for these conditions. The International Pediatric Thrombosis Network (IPTN) is a collaboration of 74 centers across 27 countries (as of January 2021) which has started important projects like the international neonatal RVT registry, while the International Pediatric Stroke Study (IPSS) group is in the planning stages of a randomized controlled trial to evaluate the utility of ACT in the management of neonatal CSVT.

## Introduction

The incidence of thrombotic disorders in children is increasing with advances in diagnostic modalities, supportive care, and management of many health conditions. There is a bimodal peak in the pediatric age group, with the highest incidence in neonates and adolescents^1^. The developing coagulation system, need for intensive care, including catheterization, and co-morbid conditions are responsible for the relatively high risk of thrombosis in neonates compared to older children^2^. Prevention and appropriate treatment are key to minimizing morbidity and balancing the adverse effects of treatment. Here, we will discuss the advances over the last 3 years in neonatal thrombosis, including epidemiology and common presentations (cerebral sinovenous thrombosis [CSVT], renal vein thrombosis [RVT], and portal vein thrombosis [PVT]). The topics of perinatal stroke and neonatal anticoagulation are not included in this manuscript.

## Epidemiology of neonatal thrombosis

### What is known?

Registry data from Canada, Germany, and Denmark have reported the incidence of neonatal thrombosis as 2.4 per 1,000 neonatal intensive care unit (NICU) admissions, 5.5 per 100,000 live births, and 4.09 per 100,000 person years, respectively^1,3,4^. The risk factors for the development of neonatal thrombosis include central arterial/venous catheterization (CAVC), sepsis, major surgery, co-morbid conditions (excluding congenital heart disease and congenital nephrotic syndrome), elevated hematocrit, and thrombophilia^4–10^. 

### What is new?

The incidence of neonatal thrombosis in the contemporary era is higher than that of registries from the 1990s and early 2000s. Siraichainan *et al*., Unal *et al*., Bhat *et al*., and El-Naggar *et al*. reported incidences of 7.3, 6.9, 15, and 15 per 1,000 NICU admissions, respectively^11–14^. The increase can be explained by advances in management and survival of younger neonates as well as improved detection of thrombosis. Additionally, two studies found that the incidence of symptomatic CAVC-associated thrombosis was 1.2% and 2.1%^15,16^.

Male sex, longer duration of CAVC (≥ 14 days), being outborn, and femoral location have been found to be additional risk factors for neonatal thrombosis. Bhat and colleagues conducted a large case-control study with 64 thrombotic neonates matched to four controls each based on catheter status and gestational age. With conditional multivariable logistic regression, male sex (odds ratio [OR] 2.12) and the previously known risk factor of sepsis (OR 3.47) remained independently associated with venous thrombosis^13^. Another study by El-Naggar *et al*. found that catheter days and being outborn (in addition to sepsis known previously) were significant risk factors in 463 neonates with venous thrombosis (from a cohort of 39,971 neonates across 30 Canadian NICUs)^14^. Dubbink-Verheij *et al*. and Lambert *et al*. reported four and seven times, respectively, increased risk of thrombosis associated with femoral location^15,16^. This finding was well supported by a 2019 paper from Barone and colleagues, who evaluated the mean diameter of multiple veins in 100 neonates weighing 500–3,000 g. The diameter of the femoral vein was ≤3 mm in all infants <2,500 g, while that of the brachiocephalic vein was consistently ≥3 mm in all weight categories^17^.

### Future direction

All but one of the incidence and risk assessment studies are small, involving fewer than 100 cases. Larger, prospective, and multicenter studies are required to delineate the most important risk factors for neonatal thrombosis. One such effort is being conducted through the International Pediatric Thrombosis Network (IPTN), which is an international registry capturing neonatal and pediatric thrombosis patient information from 74 centers in 24 countries (personal communication, January 2021)^18^.

Despite the recognition of increasing incidence and risk factors for neonatal thrombosis, there are no evidence-based strategies for prevention. Development of a risk-stratification model to identify neonates who may benefit from prophylactic anticoagulation can be useful.

## Neonatal cerebral sinovenous thrombosis

### What is known?

The reported incidence of neonatal CSVT is variable, ranging from 1–12 per 100,000 in the Dutch registry to 47 per 100,000 neonates in the Canadian registry^19,20^. Maternal and perinatal risk factors are frequently implicated, with some contribution from thrombophilia. While seizures and neonatal encephalopathy are frequent clinical signs, non-specific signs and symptoms such as poor feeding, dehydration, and sepsis can also precede the diagnosis^21^. The management of neonatal CSVT, including the use of anticoagulation therapy (ACT), remains controversial because of the lack of efficacy and safety data and increased risk of intracranial hemorrhage (ICH)^22^. There are some observational studies highlighting the safety of ACT; however, they are small and limited to single centers^23,24^.

### What is new?

Two studies reported the incidence of neonatal CSVT in the context of therapeutic hypothermia (TH) and cardiac surgery. Radicioni *et al*. demonstrated a 27% (10/37) incidence of neonatal CSVT post TH for asphyxia, which is higher than previously thought^25^. Given the overlap of signs and symptoms in these two conditions, this study subjected every newborn receiving TH to brain magnetic resonance venography (MRV) after rewarming. Claessens *et al*. found a 28% (11/40) incidence of neonatal CSVT post cardiac surgery^26^. Younger age and lower weight at surgery and the use of central venous catheter were reported risk factors.

The literature on neonatal CSVT management was strengthened by the addition of a systematic review and meta-analysis by Rossor and colleagues^27^. Their meta-analysis of limited studies (two for each outcome) showed that the use of ACT did not affect mortality and incidence or extension of ICH while reducing the risk of thrombus propagation (risk ratio 0.09, 95% confidence interval 0.02–0.47). Additionally, an analysis from the Seizures in Pediatric Stroke study group reported a 20% incidence of epilepsy at 1 year follow-up among five neonates with CSVT^28^.

### Future direction

Neonatal CSVT is a major cause of morbidity, with 40–80% of patients developing poor neurodevelopmental outcome in the long term^21^. It is unclear whether ACT can change this trajectory in the long term, or even if it is safe in the short term. There is an urgent need for a randomized controlled trial to evaluate the utility of ACT in the management of neonatal CSVT. International stroke experts are planning such a trial as part of the International Pediatric Stroke Study (IPSS) group^22^. In the meantime, consistency in managing these patients is important, as can be achieved through adopting an institutional algorithm, so we can continue to learn from our actions by ongoing evaluation of the outcome.

## Neonatal renal vein thrombosis

### What is known?

The renal vein is the most common location for non-CAVC-associated thrombosis, accounting for 10% of all neonatal thrombosis^1^. Specific risk factors include perinatal asphyxia, dehydration, and maternal diabetes^29,30^. The majority of cases (70%) are unilateral, of which two-thirds are left-sided RVT^31^. The classic triad of signs/symptoms—hematuria, flank mass, and thrombocytopenia—is present in only a small proportion, hence a high index of suspicion is necessary^32^. Despite the lack of randomized controlled trials, the commonly accepted management (based on observational studies) is supportive care without anticoagulation for unilateral RVT and anticoagulation for bilateral RVT or unilateral RVT with inferior vena cava extension^31^. Fibrinolytic therapy can also be considered in patients with bilateral RVT to increase the chance of thrombolysis and potentially reduce the risk of chronic renal failure^33–36^. With or without anticoagulation, the outcome is renal atrophy on the affected side and occurs in about 75% of cases^33^.

### What is new?

There was a population-based retrospective cohort study by Ouellette *et al*. examining the incidence, risk factors, and long-term outcomes of neonatal RVT in the province of Ontario, Canada^37^. The authors reported an annual incidence rate of 2.6 per 100,000 live births. After adjusting for gender, neonatal co-morbidities, and maternal risk factors, patients with RVT had 12.3-fold increased risk of chronic kidney disease or death and a 15.7-fold increased risk of hypertension compared to controls.

Additionally, a single-center case series of five patients was conducted in Switzerland and confirmed known risk factors and outcomes in their population^38^. It was unusual that all of their patients presented with the classic triad of signs/symptoms and RVT was right-sided in 80%. However, it is difficult to draw any conclusions because of small numbers. Another study was conducted in Poland by Mikolajczak and colleagues and reviewed the sequential ultrasound findings in neonates with RVT^39^. Along with the obvious echogenic material in the renal vein and lack of Doppler flow, they highlighted the importance of kidney enlargement, loss of cortico-medullary differentiation, and echogenic interlobular streaking as early ultrasound findings in RVT.

### Future direction

The guidelines for managing neonatal RVT are based on expert opinion and derived from small observational studies. Larger prospective studies are necessary to decipher the exact incidence of short- and long-term outcomes of neonatal RVT and whether anticoagulation helps to reduce morbidity. Through the IPTN initiative, a prospective RVT registry is one of the first multicenter studies that has been started to aid in the process^18^. In future, a multicenter randomized controlled trial in neonatal RVT management would be most helpful in providing evidence-based recommendations.

## Neonatal portal vein thrombosis

### What is known?

Neonatal PVT is rare (incidence 1/100,000 live births) and most cases are asymptomatic^40,41^. The most common risk factor for PVT is umbilical venous catheterization (UVC)^42^. While the majority of cases are isolated to the left portal vein, some can involve the right or main portal vein as well as extend to the inferior vena cava^41^. The goal of treatment is to avoid long-term complications such as portal hypertension (PHN) and gastrointestinal (GI) bleeding, although the precise incidence of and risk factors for these are unknown^43^. Secondly, it is unclear if ACT prevents these morbidities in later childhood^44^. Owing to these uncertainties, there is a lack of evidence-based guidelines for the use of anticoagulation in PVT. Two recommendations are clear: 1) remove UVC upon diagnosis of PVT (if clinically safe), and 2) if choosing supportive care, one should monitor closely with ultrasound and in case of progression to consider anticoagulation^45,46^.

### What is new?

The diagnosis and evolution of asymptomatic PVT secondary to UVC was depicted by three recent studies: Cabannes *et al*.^47^, Hwang *et al*.^48^, and Dubbink-Verheij *et al*.^49^. Similar to previous reports^50–52^, these studies showed a wide range of incidence from 21.7% in Hwang *et al*. to 75% in Dubbink-Verheij *et al*. The majority of PVT resolved spontaneously (97.2% in Cabannes *et al*.’s study and 100% of followed patients in Dubbink-Verheij *et al*.’s study at 1 year; Hwang *et al*.’s study had poor follow-up) without treatment. These data support the previously published recommendation to treat PVT conservatively without anticoagulation as long as UVC can be removed and there is no progression to multiple portal veins and/or inferior vena cava^36,40^.

Our group published a retrospective study looking at the outcomes of 74 premature and term neonates with symptomatic PVT^53^. One-third of this cohort had residual thrombosis at median follow-up of 16.6 months (40.5% were followed for <6 months at the time of publication). The difference can be explained by reduced follow-up duration in a cohort of patients and possible increased severity of thrombosis. The incidence of symptomatic PVT was 8.4 per 1,000 NICU admissions in our cohort, much less compared to 36 per 1,000 admissions reported by Morag and colleagues^42^.

The long-term risk of PHN and its complications (esophageal varices and GI bleeding) are the most dreaded consequences of neonatal PVT. Although the exact incidence is unknown, it seems to occur in a small number of cases (4.5% in one study of 133 neonates)^42^. Di Giorgio and colleagues published a national survey of patients with PHN secondary to PVT, highlighting the significant morbidity faced by these patients^54^. They presented data on 187 children with PHN diagnosed at mean age of 4 ± 3.7 years. One-third (36%) of patients experienced GI bleeding, and 87% of those who had endoscopy were diagnosed with esophageal varices. After a mean follow-up of 11.2 ± 4.8 years, 34% of children required surgical intervention. These results re-emphasize the need for larger studies to determine the risk factors for the development of PHN among patients with neonatal PVT. This will help to devise possibly more aggressive prophylactic and therapeutic management strategies in the neonatal period and improved surveillance beyond. For surveillance, this study highlights the need to monitor beyond the 5 years suggested in the literature^41^.

### Future direction

As discussed above, there is an urgent need for larger prospective studies to elucidate the risk factors for long-term PHN in neonates with PVT. Once these are known, it can help to improve management and surveillance strategies for those at risk.

## Conclusion

The burden of thrombotic disorders in neonates is increasing with time, in part because of improvements in the survival of younger newborns and advances in medical care. The data on the incidence, risk factors, management, and outcomes of these disorders are currently derived from small, mostly single-center, observational studies. Collaboration among thrombosis experts is urgently needed to advance this field and in turn improve the lives of our vulnerable newborns. It is with great hope that we look toward groups like IPTN (https://www.isth.org/page/iptn) and IPSS (https://internationalpediatricstroke.org/ipss-research) to lead the way in this effort. We encourage centers to consider joining these groups in an effort to enhance research and knowledge in this field.
